# Immunohistochemical Evaluation of Bone Remodeling Following Compressive Force on Mandibular Condyle

**DOI:** 10.3390/biology14030228

**Published:** 2025-02-23

**Authors:** Ioannis Lyros, Ioannis A. Tsolakis, Georgia Kotantoula, Konstantinos Tosios, Vilaras George, Nikolaos Nikitakis, Efstratios Ferdianakis, Theodoros Lykogeorgos, Eleni Fora, Apostolos I. Tsolakis

**Affiliations:** 1 Department of Orthodontics, School of Dentistry, National and Kapodistrian University of Athens, 11527 Athens, Greece; geokotantouladds@yahoo.com (G.K.); stratis-fer@hotmail.com (E.F.); apostso@otenet.gr (A.I.T.); 2 Department of Orthodontics, School of Dentistry, Aristotle University of Thessaloniki, 54623 Thessaloniki, Greece; tsolakisioannis@gmail.com; 3 Department of Orthodontics, Case Western Reserve University, Cleveland, OH 44106, USA; 4 Department of Oral Medicine & Pathology and hospital Dentistry, School of Dentistry, National and Kapodistrian University of Athens, 11527 Athens, Greece; ktosios@dent.uoa.gr (K.T.); nnikitakis@dent.uoa.gr (N.N.); foraeleni@gmail.com (E.F.); 5 Department of Pathology, Medical School, National &Kapodistrian University of Athens, 11527 Athens, Greece; vilarasgeorge@gmail.com; 6 “Hatzikosta” General Hospital of Messolonghi, 30200 Messolonghi, Greece; theolyk@gmail.com

**Keywords:** mandibular posterior displacement, orthodontics, condyle, RANK ligand, TRAP, immunohistochemistry, remodeling

## Abstract

Excessive mandibular growth is largely affected by genetic factors and its orthodontic management is challenging. The present study utilized histophotometry and aimed to investigate immunohistochemical alterations in rat condyles following posterior mandibular displacement using a fixed intraoral functional appliance. Seventy-two male Wistar rats were divided into two equal groups, experimental and control, each consisting of three equal subgroups. The animals were sacrificed after 30, 60, and 90 days, and their condyles were isolated and examined microscopically for potential immunohistochemical changes. Statistically significant differences in RANKL appeared at days 30 and 60, while noteworthy changes regarding TRAP were evidenced at days 30 and 90. In the experimental group, RANKL decreased statistically significantly between days 60 and 90, while in the control group, the RANKL measured significantly increased at day 90 compared to days 30 and 60 alike. In the experimental group, TRAP appeared significantly decreased at day 60 vs. 30, while at day 90 vs. 60, it was found to be significantly increased. In the control group, there were no significant changes in TRAP. Posterior mandibular displacement may cause immunohistochemical changes in the rat condylar bone, and such outcomes should be considered when planning similar procedures in humans.

## Simple Summary

Extreme mandibular growth makes people seek orthodontic treatment in order to manipulate their appearance. Orthodontic treatment often includes intraoral devices to face the skeletal problem. This experiment used healthy male rats and repositioned their mandibles backwards. Immunohistochemical changes in condyles, related to bone metabolism, revealed some statistically significant differences, and also some noteworthy local microscopic alterations of the condyle were evidenced. Remarkable differences in RANKL appeared at days 30 and 60, and changes regarding TRAP became important at days 30 and 90. In the experimental group, RANKL decreased significantly between days 60 and 90, while in the control group, the moleculewas found significantly increased at day 90 compared to days 30 and 60. In the experimental group, TRAP dwindled significantly at day 60 vs. day 30, while at day 90 vs. 60, it appeared significantly elevated. In the control group, there were no statistically important alterations in TRAP. In sum, specially selected biomarkers like these included in the present study can explain the localized biochemical turmoil resulting in architectural changes occurring throughout functional guidance of the lower jaw.

## 1. Introduction

The 2019 Policy Statement of the World Dental Federation highlights the importance of dental alignment and facial symmetry for human appearance and self-esteem, advocating to inform the public about the condition and the provision of care under the supervision of the orthodontist or a qualified, properly trained, dental practitioner [1]. A relatively high rate of malocclusion has been reported in preschoolers, and it seems to be associated with deleterious oral habits [2,3]. The prevalence of occlusal discrepancies varies significantly between different child and adolescent populations [4,5,6] and the various traits might be affected by intraoral conditions [4,7], ethnicity [7,8], or sociodemographic issues [4,9,10].

In skeletal class III malocclusion, a discrepancy regarding the anteroposterior intermaxillary relationship is often registered. The mandible may appear protruding, the maxilla can appear underdeveloped, or a combination of both conditions might be encountered [11]. The prevalence of the condition exhibits variability between studies [8]. Affected by complex genetic factors [12,13], it varies between human races [13], throughout the period of growth and the consecutive phases of the developing dentition [8]. Currently, there are three approaches for preventing or alleviating mandibular prognathism, growth modification at a younger age, orthodontic camouflage treatment, and orthognathic surgical correction [12].

Considering the challenge, during the period of growth, the orthodontist may seek to decrease the rate of mandibular growth by implementing devices exerting retracting force on the lower jaw [14]. Posterior mandibular displacement affects mandibular morphology [15] and promotes the development of a smaller mandible at a grown age, likely producing localized histological variations in condylar and bone structure [16].

Laboratory animal testing has been popular in bone research investigating growth and tissue remodeling [17,18]. There is a long history and experience of using rats to study mandibular and condylar growth [19,20,21]. Due to public awareness of animal welfare and growing concern for their use in experiments [18,22], there are strict regulations regarding the protocol and the procedure. The rat is most commonly preferred for research on cranial growth due to the relative easiness of reproduction and breeding [17], and the existing overall anatomical and histological similarities with humans [23].

Bone tissue histology is commonly evaluated with light microscopy after sample staining. Descriptive histology provides a comprehensive assessment of the organ under study, giving information on the morphology and structure of its tissues, as well as the characteristics of their cells and extracellular substance. Conversely, histomorphometry implements a measurable analysis of histological data, namely the dimensions, area, and amount of the elements of interest [24,25]. Despite efforts to increase reliability, histological and histomorphometric evaluation may still be prone to biased conclusions due to methodological inaccuracies and inter-observer variability [26,27].

Immunohistochemistry (IHC) is used to identify peptidic molecules of interest in sections of tissue, based on the principle of the specific interaction between antigens and antibodies [28]. To increase IHC outcome reliability and reproducibility, quantitative methodologies have been developed, relying upon digital analysis using dedicated computer software [26,28,29,30].

Resorption is an essential aspect of bone development and remodeling [31]. The predominant function of the osteoclasts in this process [32] and the significance of their interaction with the osteoblasts [33] have been acknowledged. Originating from hematopoietic stem cells, large multinucleated osteoclasts are responsible for bone resorption [32]. Initially, common myeloid progenitors are stimulated by granulocyte/macrophage stimulating factor (MCSF) to differentiate into granulocyte/macrophage progenitors, which transform into monocytes/macrophages to become osteoclast precursors that eventually enter the blood circulation, migrating to immature bone, becoming multinucleated [34]. Osteoblasts secrete MCSF that allows for survival, proliferation, differentiation, and mobility of matured osteoclasts [33]. RANKL (Receptor Activator of Nuclear factor Kappa B ligand), a Tumor Necrosis Factor family memberand also an Osteoprotegerin (OPG) ligand, is highly expressed in osteoblasts and osteocytes and binds to its specific receptor (RANK), on the surface of osteoclasts and osteoclast precursors, leading to osteoclast differentiation, fusion, and activation [33]. The circuit of RANKL/OPG promotes or suppresses osteoclast differentiation and proliferation [32].

TRAP (tartrate-resistant acid phosphatase) is an enzyme commonly, but not exclusively, identified in the osseous tissue of humans and rodents. It is expressed by macrophages and osteoclasts and has been used as a histochemical marker of osteoclastic activity although it has also been implicated in osteoblastic regulation. TRAP is secreted by the osteoclast during bone resorption. Therefore, abnormal activity is registered in osteoporosis, hyperparathyroidism, rheumatoid arthritis, and neoplasms. TRAP 5b, a serum isoform, is the predominant form secreted by the osteoclast. TRAP is important for collagen type I (the predominant protein in the bone matrix) production and degradation [35].

The present study utilized immunohistochemical analysis and histophotometry to trace and quantify TRAP and RANKL, as markers of osteoclastic activity, in the trabecular bone of rat mandibular condyles, which had been displaced posteriorly using a cemented orthodontic intraoral functional appliance. The findings may enhance our understanding of systemic and regional disturbances in animals and could potentially be extrapolated to clinical practice for human patients undergoing orthodontic interventions to correct developmental discrepancies.

## 2. Materials and Methods

Ethical approval of the experimental protocol (number 598742/04-10-2019, registered as EL 25 BIO 05) was obtained from the Veterinary Directorate according to the Greek national legislation (P.D 56/2013), conforming to the rules of the European Directive 2010/63/EE and the European Council (276/33/20.10.2010) that refer to the protection of vertebrate experimental animals used in scientific research.

### 2.1. Experimental Design

In the current study, seventy-two (72) four-week-old male Wistar rats were recruited. After an initial four-week breeding period at the Hellenic Pasteur Institute, they were transferred and housed at the Laboratory for Experimental Surgery and Surgical Research “N. S. Christeas” located at the University Laboratory, School of Medicine, Athens, Greece. Animal cages (Tecniplast S.P.A., Italy) were selected to follow National and European legislation. Centrally ventilated (15 air changes per hour), they remained in stable conditions of relative humidity (55%), temperature (20°C ± 2°C), and artificial lighting (alternating 12h cycles of light and dark). Access to food and water was offered without restriction. Throughout the entire period of study, all rats underwent close monitoring to ensure proper growth and development.

Equal and random animal grouping, to be called group A (experimental) and B (control), was performed with the aid of Random Team Generator, a free online tool. Next, each group was sub-divided into three equally sized subgroups comprising twelve rats each, in respective chronological sequence (A1, A2, A3 versus B1, B2, B3).

**Figure 1:**
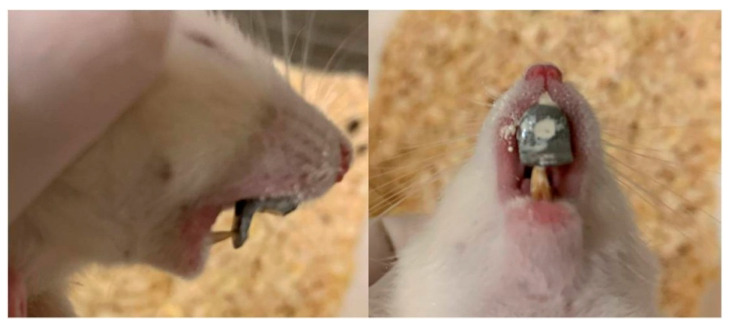
The modified orthodontic intraoral device cemented to the maxillary incisors.

A modified, previously described [36], fixed, functional intraoral orthodontic appliance was cemented to the experimental animal maxillary incisors with the aim to promote posterior mandibular displacement (Figure 1). Full metal casts had been priorly lab-developed, their fabrication having been based on earlier digital intraoral scanning (TRIOS 3, 3Shape intraoral scanner, 3Shape, Inc., New Providence, NJ 07974, United States of America) of a rodent that had been picked at random. Cementation was accomplished using zinc phosphate cement (Harvard Cement Normal Setting; Harvard Dental International GmbH, Hoppegarten, Germany).

In the course of the study, all animals indiscriminately were provided with mashed food, namely well-defined proportions of pellets mixed with water in oatmeal-like consistency.

Overall, experimental procedures and relative screening were finished by day 90. Animal euthanasia was actualized on day 30 (subgroups A1, B1), day 60 (subgroups A2, B2), and day 90 (subgroups A3, B3). On experimental day 60, orthodontic appliances were debonded from rats still remaining in the experimental subgroup A3.

### 2.2. Immunohistochemical Analysis

Animals were anesthetized by intramuscular injection of a ketamine–xylazine solution (0.2 mL/kg). Next, they were sacrificed, their heads to be dissected and the soft tissues to be gently removed. All animal surgical procedures were performed at the Laboratory for Experimental Surgery and Surgical Research “N. S. Christeas” at the University Laboratory, School of Medicine, Athens, Greece, in line with the requirements established by the Greek State [37,38] and the European Code for the Care and Use of Animals for Scientific Purposes [39]. Each head intact and separately was fixed in 10% formalin solution.

**Figure 2:**
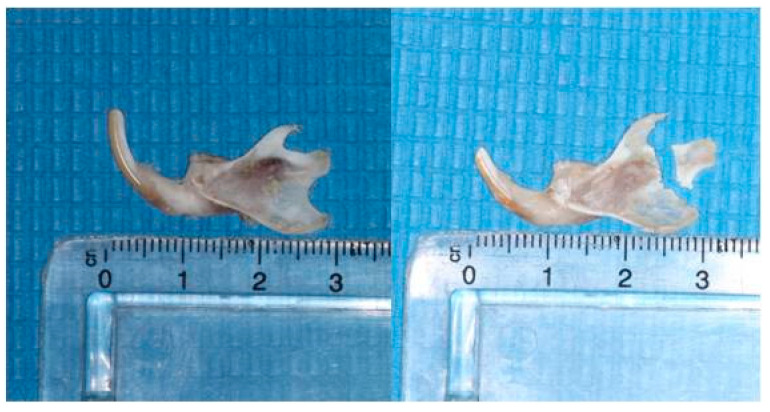
Isolation of the mandibles and condyles.

Heads were cut in the middle and the left mandibular condyles were isolated to be immersed in ethylene diamine tetra-acetic acid solution (MICRODEC EDTA-BASED, DIAPATH S.p.A) for 10-day decalcification (Figure 2). Subsequently, the condyles were embedded in paraffin using conventional methods and a fully motorized rotary microtome (ARM3600, Histo-Line Laboratories Co., Pantigliate, Italy) was used to create 6 *μ*mthick serial sections parallel to the sagittal condylar plane. The sections were stained with antibodies to observe potential immunohistochemical activity.

Immunohistochemical analysis aimed to identify and localize the expression of proteins RANKL and TRAP related to bone remodeling, utilizing the following primary antibodies: rabbit polyclonal RANKL (dilution 1:100, Catalog No. E-AB-60724, Elabscience©, Elabscience Biotechnology Inc., Houston, TX, USA) and mouse monoclonal TRAP (dilution 1:100, Catalog No. PA6219, Elabscience©). Immunohistochemical staining was performed with the BenchMark ULTRA IHC/ISH System (Roche Tissue Diagnostics, Roche Diagnostics, Ltd., Rotkreuz, Switzerland) according to accredited staining protocols. Sections have been post-stained with hematoxylin. Positive staining appeared as brown cytoplasmic color.

Stained sections were scanned at x10 magnification with an Olympus CX23 RFS2 microscope (Olympus, Tokyo, Japan) equipped with a digital camera (Leica DC300F, Leica Microsystems AG, Heerbrugg, Switzerland) and Sedeen Viewer© software (version 5.4.4, Pathcore Inc., Toronto, ON, Canada) was implemented to convert produced TIFF files into JPEG images for digital analysis.

**Figure 3:**
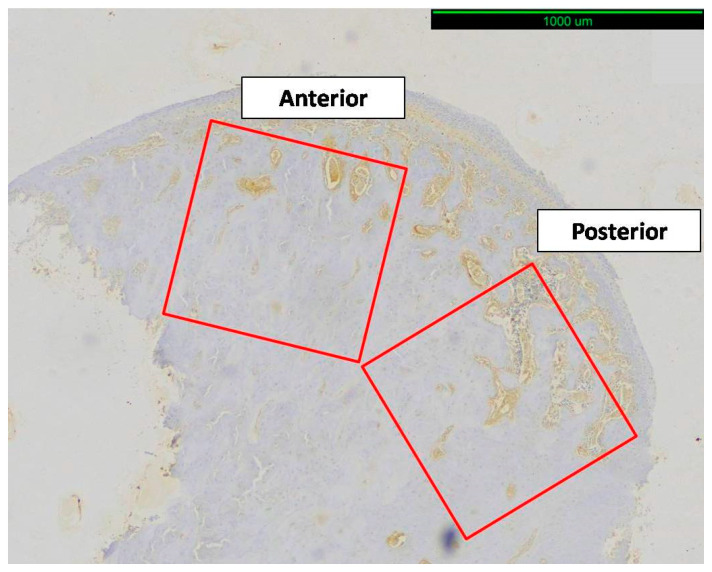
Immunohistological image of sagittal sections of condyle. Square condylar head regions of interest (anterior and posterior), measuring approximately 1.0 × 1.0 mm (original magnification ×10).

**Figure 4:**
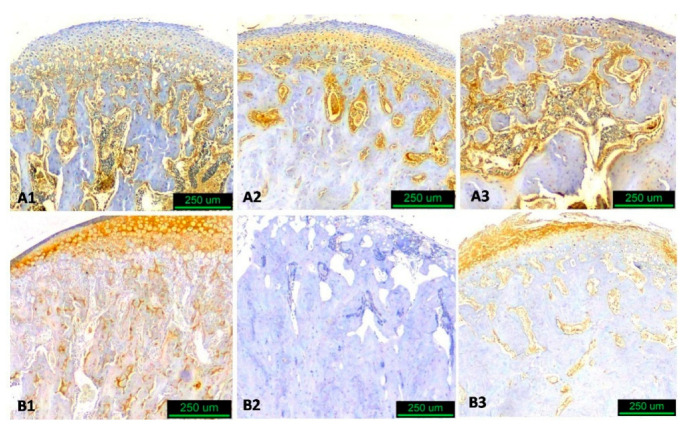
Immunohistological images of sagittal sections of condyle at three different observation times. Group A experimental, Group B control 1 = 30 d, 2 = 60 d, 3 = 90 d. (RANKL staining—hematoxylin counterstain, original magnification ×10).

**Figure 5:**
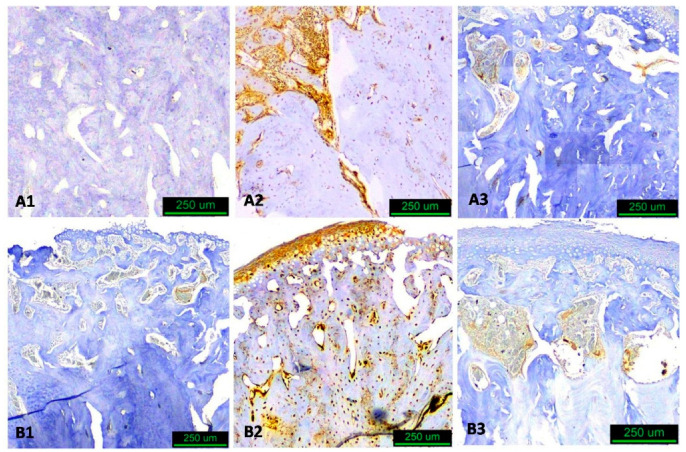
Immunohistological images of sagittal sections of condyle at three different observation times. Group A experimental, Group B control 1 = 30 d, 2 = 60 d, 3 = 90 d. (TRAP staining—hematoxylin counterstain, original magnification ×10).

To evaluate the expression of RANKL and TRAP in the condylar subchondral trabecular bone, i.e., trabecular bone underlying the condylar cartilage, two squares measuring approximately 1.0 mm × 1.0 mm (anterior and posterior regions of interest) that enclosed only trabecular bone were selected. The brown-colored area/total area ratio was calculated (Figure 3, Figure 4 and Figure 5) with the digital tool Image-Pro Plus© (version 6.0.0.260, Media Cybernetics, Inc., Rockville, MD, USA), which converted the results to μm^2^. For each animal, the mean value of the anterior and posterior regions was used for statistical analysis.

### 2.3. Statistics

Sample size was calculated by performing power analysis. Ethical reasons enforced kept the number of animals sufficiently small, but sufficient to reliably expose any potential statistically significant outcomes. The potential for some animals to experience stress or adverse reactions during the experiment was considered.

Finally, 72 animals in total and subgroups of 12 subjects each were decided, using standard statistical criteria (a = 0.05, b = 0.10), yielding a power of 90%to detect 0.5 mm difference (26.5 vs. 27.0 SD 0.37) for the primary outcome of the study, namely mandibular length, as being previously mentioned [15], equally divided into experimental and control groups.

Differences in mean RANKL and mean TRAP expression between methods and time (30, 60, and 90 days), were investigated using generalized linear models (GLM) with logit link, binomial family, and robust standard errors, since the outcome variables were expressed as percentages. Independent variables in the models were the method, the time of intervention, and their interaction. Bonferroni correction for multiple comparisons was applied in the posthoc test.

All tests were two-sided, and level of statistical significance was set at a = 5%. Stata v13 was used for the statistical analysis (StataCorp LP., College Station, TX, USA).

## 3. Results

Descriptive characteristics, in terms of the mean, standard deviation (SD), and minimum and maximum of mean RANKL (%) and mean TRAP (%) expression, are presented in Table 1.

**Figure 6:**
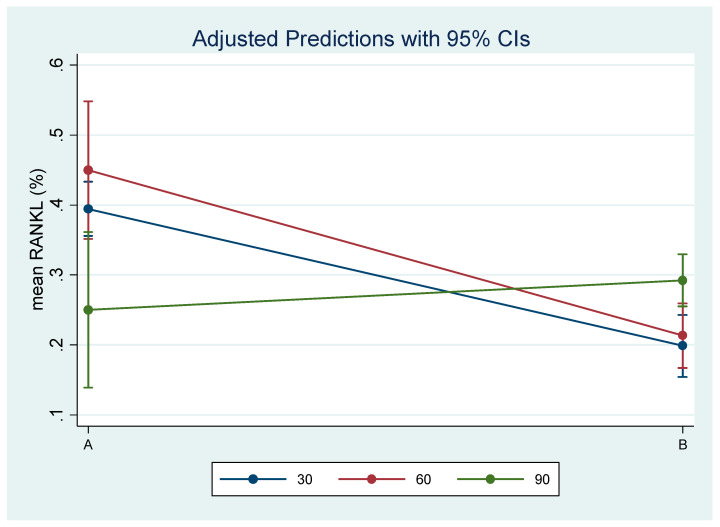
Estimated mean RANKL (%) expression and 95% Confidence Interval by time and treatment.

**Figure 7:**
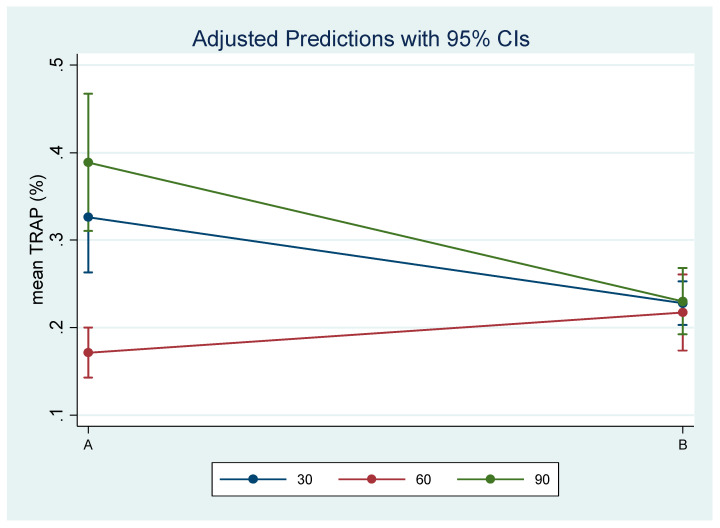
Estimated mean TRAP (%) expression and 95% Confidence Interval by time and treatment.

Results are shown in Table 2 and Table 3 and Figure 6 and Figure 7, as the mean and 95% Confidence Intervals (95% CI). Statistically significant differences in the RANKL expression between groups were found at days 30 and 60, while in TRAP expression they were found at days 30 and 90 (Table 2, Figure 6 and Figure 7). Specifically, RANKL expression was found statistically significantly increased in experimental group Aat days 30 and 60 compared to control group B. TRAP expression showed a statistically significant increase at days 30 and 90 in experimental group A.

A statistically significant decrease in RANKL expression atday 90 vs. 60 was found for experimental group A, while in control group B, RANKL expression was increased significantly at day 90 compared to both 30 and 60 (Table 3, Figure 6).

TRAP expression was significantly decreased at day 60 vs. 30, while at day 90 vs. 60, it was found to be significantly increased for experimental group A. In control group B, there were no changes in TRAP expression (Table 3, Figure 7).

The conservative properties of the Bonferroni correction method for multiple comparisons should be taken into account in the interpretation of the results.

## 4. Discussion

Facial skeletal discrepancy is a challenge for the clinical orthodontist, who is summoned to correct malocclusion and guide facial proportions into harmony [28]. Patients seeking orthodontic rehabilitation usually experience discomfort regarding their image, coupled with dental irregularity [40]. Therefore, the treatment plan aims to establish skeletal balance and boost the self-esteem of the patient.

Individuals already exhibiting a tendency or having skeletal class III malocclusion may need to undergo mandibular posterior displacement, which is intimately related to bone remodeling [14,15,16,41]. To unveil details of the process, an experiment was conceived and run with the recruitment of lab animals, namely Wistar rats. The rat has been popular in developmental issue research [21,42] despite anatomical and physiological differences that might produce inconsistencies when validating the observations before extrapolating observations onto humans [43].

The bonding of the intraoral device used in the current study and animal sacrifice were accomplished while the animals were being held unconscious. A properly prepared solution of ketamine–xylazine was the anesthetic medication used for the above experimental procedure. All rodents were kept under close monitoring throughout the entire experimental period for potential unwellness or symptoms of stress. Test animals continued to gain weight as had been anticipated up to the conclusion of the study, despite the introduction or removal of the intraoral device.

Rats were selected to be all male and of the same age for reasons of sample homogeneity, as has been previously suggested by others [44,45]. However, their gender might confound present measurements of interest that are affected by hormones in growing subjects. Thus, further research involving female animals could prove informative. Animal breeding and their living conditions were kept strictly standardized.

Bone metabolism features a close interaction of mesenchymal cells such as osteoblasts, osteoclasts, and osteocytes that function in harmony to preserve the volume and the integrity of the ossified structure [46]. Assays for biochemical markers, reflecting osteoblastic and osteoclastic enzymatic activity within the bone, provide insight intocomplex metabolic pathways culminating in macroscopic structures.

The present immunohistochemical investigation implemented proper biological tools to reach conclusions on the remodeling of the mandibular condyle after the application of functional tension, similar to what may happen in daily practice when treating orthodontic patients with pronounced developmental enlargement of the lower jaw. A rabbit recombinant human RANKL polyclonal antibody and a mouse TRAP monoclonal antibody were implemented to increase our understanding of the timing and the degree of localized mandibular alterations when activating functional appliances to address excessive, disproportional, undesirable facial growth. Allegedly, the aforementioned therapeutic interventions are unlikely to induce severe systemic disruption in humans within established guidelines of practice [16].

Bone undergoes constant remodeling throughout a lifetime. It includes various components, cells such as osteoclasts, osteoblasts, and osteocytes embedded in an extracellular matrix, composed of organic and inorganic molecules. The bone microenvironment is constantly changing, affected by factors such as aging, lifestyle, health conditions, nutritional status, and pathology. Bone cells constantly undergo metabolic adaptation to counter these changes [47].

Tightly regulated, bone remodeling is affected by metabolic hormones [48] and the availability of nutrients [49]. Osteoclasts are multinucleated cells resorbing bone and have a myeloid origin. MCSF, an essential growth factor for the differentiation and survival of myeloid cells and RANKL, is a key factorin osteoclast differentiation and function [50]. The RANKL/Osteoprotegerin (OPG) system is essential for bone resorption.

Human and rodent RANKL have 85% common amino acid sequences. RANKL belongs to the TNF cytokine superfamily.OPG, a cytokine receptor of the TNF receptor superfamily, shares an important role in bone metabolism as receptor for RANKL in the RANKL/OPG axis, inhibiting the emergence of osteoclasts and the ensuing bone resorption. Mature osteoclasts localizein the bone and release bone-resorbing enzymes. When bone resorbs, collagen and minerals are released to create space and minerals used by osteoblasts to produce new bone [51].

TRAP, an iron-containing enzyme, is found in humans and murine species [47]. TRAP serum levels reflect the size of the osteoclast population and bone remodeling activity. As a consequence, TRAP is an established marker of osteoclastic population. TRAP may have a role in sex-connected bone mineralization. Therefore, TRAP exerts important functions in skeletal development and maintenance [52].

In the present immunohistochemical animal study, statistically significant differences were evidenced regarding the expression of RANKL and TRAP between the experimental and control groups. Increased RANKL expression between experimental subgroups A1 (30 days with the device in place) and A2 (60 days with the device in place) and control subgroups B1 and B2, respectively, depict the ongoing bone remodeling, in essence showing bone resorption triggered by compressive functional force. Such differences seem to fade at 30 days after the removal of the appliance (day 90 of the experiment), as highlighted by the statistically non-significant difference between experimental subgroup A3 and control subgroup B3. The statistically significant increase in TRAP expression of experimental subgroup A1 in relation to control group B1 supports the suggestion that resorption predominates in bone remodeling. Interestingly, TRAP expression also appears statistically significant even at 90 days of the experiment, namely 30 days after the removal of the intraoral apparatus.

According to the measurements presented in Table 3, it might be suggested that bone resorption happened throughout the experiment in the animals designatedas test (group A) and control (group B) alike. These outcomes mean that the rats seem to follow a continuous path of growth and their mandibles remodel independently, definitely not utterly restricted by the scheduled intervention.

It is noteworthy that past research has implemented asimilar intraoral apparatus to achieve distal mandibular displacement before investigating ensuing immunohistochemical changes, havingattributed them to the resulting functional malocclusion. In summary, Teramoto et al. (2003) [53] showed a reduction in the thickness of mandibular condylar cartilage and pronounced immunostaining at its anterior region featuring large hydrophilic proteoglycan molecules after functional, persistent compressive force for 3 days of experimentation, with the subsequent recovery becoming evident at 7 days at the end of functional retraction [51].

Cholasueksa et al. (2004) [44] studied the immunohistochemical changes of nerve fibers in male Wistar rats. A generic marker for the neural tissue, namely protein gene product 9.5 (PGP 9.5)—immunoreactive (IR) nerve fibers, was examined in lateral site portions of the articular discs, posterior disc attachments, joint capsules, and synovial membranes. However, in the central disc regions, nerve fibers were undetectable. In the control group, growth-associated protein-43 (GAP-43)—IR nerve fibers localized only around blood vessels, while in the experimental group, the above nerve fibers were also present posteriorly, at disc attachments and the synovial membrane. The GAP-43–IR nerve fibers ceased to exist after the end of growth and in cases lacking sympathetic nerve fiber injury [44].

Lastly, the conclusion reached by Figueroba et al. (2014) [54] is in agreement with the result of the present study because they found that the functional posterior displacement of the mandible increased the level of local inflammation and induced alterations in the condylar shape [54].

Different appliances have been used in animal experiments for posterior forced mandibular traction [20,53,55] or posterior mandibular guidance [36,44,54,56,57]. Heavy and light orthopedic forces were applied by Tsolakis [20] and classified based on the lateral pterygoid muscle’s response to the force. In Tsolakis’ [20] experiment, the intention was to apply force directly on the condyle and test the biological rationale underlying the use of a chin cap appliance. In the present experimental study, the appliance used did not apply any force directly on the condyle. The appliance was similar to that of Desai (1996) [36], exerting physiological muscular forceon the mandible, keeping it backwards, rather than applying direct force on the condyle. The intention was to produce a backward functional guidance of the mandible and test the rationale underlying the use of functional force in mandibular growth control. In rodents, where gnawing movements result in anterior forward movement of the lower jaw, posterior guidance of the mandible may affect its growth.

The results of the current study should be taken into consideration with much prudence, because they refer to an animal study, notwithstanding that the rat remains the most popular experimental animal model for studying human disorders [14,21,41,58,59]. The significance of the presented outcomes could be enhanced by corroboration through a longitudinal long-lasting follow-up of the models of study.

## 5. Conclusions

In the present study, functional backward mandibular repositioning in rats, produced by a cemented intraoral device, affected the expression of RANKL and TRAP, proteins involved in osseous remodeling, resulting in changes of the microarchitecture of the condylar trabecular bone. It was found that

- In experimental group A, RANKL increased statistically significantly compared to control group B after both 30 and 60 days.
- In experimental group A, TRAP showed a statistically significant increase after 30 and 90 days alike.
- In experimental group A, a statistically significant reduction in the RANKL value on day 90 compared to day 60 was found, while in control group B, RANKL increased significantly on day 90 compared to both days 30 and 60.
- In experimental group A, TRAP significantly declined on day 60 compared to day 30, while by day 90, as compared to day 60, it significantly increased. By contrast, control group B registered no significant changes in TRAP.
- A future well-conceived randomized clinical trial may be more capable of producing more reliable outcomes and sharing more robust suggestions.

## Figures and Tables

**Table 1:** Descriptive characteristics for RANKL (%) and TRAP (%) expression by treatment and time.

	Experimental Group		Control Group	
	Mean (SD)	Min–Max	Mean (SD)	Min–Max
30 days				
RANKL (%, mean)	39.45 (7.11)	(29.32–48.95)	19.85 (8.15)	(9.16–31.01)
TRAP(%, mean)	32.63 (11.59)	(16.96–47.32)	22.77 (4.55)	(16.34–33.52)
60 days				
RANKL (%, mean)	44.99 (17.99)	(14.03–67.70)	21.31 (8.42)	(9.21–38.70)
TRAP(%, mean)	17.14 (5.25)	(9.35–24.22)	21.71 (7.99)	(10.81–36.77)
90 days				
RANKL (%, mean)	25.00 (20.41)	(7.59–66.80)	29.21 (6.80)	(22.36–41.39)
TRAP(%, mean)	38.89 (14.38)	(20.99–64.76)	23.00 (6.95)	(10.81–35.77)

**Table 2:** Estimated mean difference (95% Confidence Interval) in RANKL (%) and TRAP (%) expression by treatment, per time.

	Mean Diff.	95% CI *	*p*-Value *
RANKL			
B vs. A, 30 days	−19.60	(−26.80, −12.40)	**<0.001**
B vs. A, 60 days	−23.68	(−36.91, −10.44)	**<0.001**
B vs. A, 90 days	4.21	(−10.12, 18.54)	>0.999
TRAP			
B vs. A, 30 days	−9.85	(−18.15, −1.55)	**0.013**
B vs. A, 60 days	4.58	(−1.79, 10.95)	0.256
B vs. A, 90 days	−15.89	(−26.54, −5.25)	**0.001**

* Bonferroni adjusted. Bold *p*-values indicate statistical significance at 5% level.

**Table 3:** Estimated mean difference (95% Confidence Interval) in RANKL (%) and TRAP (%) expression by time, per treatment.

	Mean Diff.	95% CI *	*p*-Value *
RANKL			
60 vs. 30 days, A	5.54	(−7.91, 18.99)	>0.999
90 vs. 30 days, A	−14.44	(−29.47, 0.58)	0.065
90 vs. 60 days, A	−19.98	(−38.90, −1.07)	**0.033**
60 vs. 30 days, B	1.46	(−6.68, 9.61)	>0.999
90 vs. 30 days, B	9.37	(1.99, 16.74	**0.006**
90 vs. 60 days, B	7.91	(0.38, 15.43)	**0.035**
TRAP			
60 vs. 30 days, A	−15.49	(−24.34, −6.64)	**<0.001**
90 vs. 30 days, A	6.26	(−6.58, 19.11)	0.893
90 vs. 60 days, A	21.75	(11.11, 32.40)	**<0.001**
60 vs. 30 days, B	−1.06	(−7.45, 5.33)	>0.999
90 vs. 30 days, B	0.22	(−5.55, 6.00)	>0.999
90 vs. 60 days, B	1.28	(−6.08, 8.65)	>0.999

* Bonferroni adjusted. Bold *p*-values indicate statistical significance at 5% level.

## Data Availability

The data presented in this study are available upon request from the corresponding author.
